# Phenotypic Plasticity Influences the Size, Shape and Dynamics of the Geographic Distribution of an Invasive Plant

**DOI:** 10.1371/journal.pone.0032323

**Published:** 2012-02-27

**Authors:** Jean-Baptiste Pichancourt, Rieks D. van Klinken

**Affiliations:** CSIRO Ecosystem Sciences – EcoScience Precinct, Dutton Park, Queensland, Australia; Max Planck Institute for Chemical Ecology, Germany

## Abstract

Phenotypic plasticity has long been suspected to allow invasive species to expand their geographic range across large-scale environmental gradients. We tested this possibility in Australia using a continental scale survey of the invasive tree *Parkinsonia aculeata* (Fabaceae) in twenty-three sites distributed across four climate regions and three habitat types. Using tree-level responses, we detected a trade-off between seed mass and seed number across the moisture gradient. Individual trees plastically and reversibly produced many small seeds at dry sites or years, and few big seeds at wet sites and years. Bigger seeds were positively correlated with higher seed and seedling survival rates. The trade-off, the relation between seed mass, seed and seedling survival, and other fitness components of the plant life-cycle were integrated within a matrix population model. The model confirms that the plastic response resulted in average fitness benefits across the life-cycle. Plasticity resulted in average fitness being positively maintained at the wet and dry range margins where extinction risks would otherwise have been high (“*Jack-of-all-Trades*” strategy *JT*), and fitness being maximized at the species range centre where extinction risks were already low (“*Master-of-Some*” strategy *MS*). The resulting hybrid “*Jack-and-Master*” strategy (*JM*) broadened the geographic range and amplified average fitness in the range centre. Our study provides the first empirical evidence for a *JM* species. It also confirms mechanistically the importance of phenotypic plasticity in determining the size, the shape and the dynamic of a species distribution. The *JM* allows rapid and reversible phenotypic responses to new or changing moisture conditions at different scales, providing the species with definite advantages over genetic adaptation when invading diverse and variable environments. Furthermore, natural selection pressure acting on phenotypic plasticity is predicted to result in maintenance of the *JT* and strengthening of the *MS*, further enhancing the species invasiveness in its range centre.

## Introduction

Understanding the mechanisms underlying the size, shape and dynamics of species geographic distributions remains a key challenge in ecology [1], [2], [3], [4], [5], [6], [7], [8], [9], [10], [11]. Phenotypic plasticity [12], [13], [14], which is the ability of a genotype to express different phenotypes in different environments, has long been suspected to be a key mechanism for allowing species to have and adaptively maintain broad geographic distributions without the need for local genetic differentiations [11], [15], [16], [17], [18]. It is also expected to allow species to more effectively shift their geographic range in response to changing climate [17], [19] and to allow invasive species to increase their ability to rapidly invade across large environmental gradients [11], [20], [21], [22], [23], [24]. However, despite strong theoretical support, the role of plasticity in influencing species geographic distributions and invasions has not yet been demonstrated in the field [11], [16], [17], [22].

Organisms' morphology can plastically respond to changes in environmental conditions, such as through independent plastic traits (such as changing plant size) or through reallocations between pairs of traits (such as between seed mass and seed number) [23], [25], [26], [27]. These plastic responses can enhance physiological (e.g., growth, germination) and demographic (e.g., survival, fecundity) components of fitness [20], [21], [28]. However, these benefits do not necessarily translate into a higher intrinsic rate of natural increase or “average fitness” [22], [26], [29], [30]. *Average fitness* is a single life-cycle measure that synthesizes the fitness benefits of all the vital components of fitness (survival, growth and fecundity) for each life-stage [29], [31], [32], [33]. There are a few examples where plastic responses have been demonstrated to increase average fitness at a local scale [26], [30]. Also, some studies have described plastic responses across species geographic ranges [15], [20], [22], but their associated average fitness benefits at such a scale, and therefore the consequences for species geographic distributions, have not yet been determined [17], [22].

Three spatial strategies have been proposed by which phenotypic plasticity can benefit components of fitness (the vital rates) for some life-stages [20] and in some cases ultimately increase average fitness across the life-cycle [22], [24]: the “*Master-of-Some*” (*MS*) strategy, where plasticity results in an increase in average fitness in a limited number of habitats or climatic conditions (fitness maximization), possibly allowing for higher population densities under favourable conditions; the “*Jack-of-all-Trades*” (*JT*) strategy, where plasticity maintains average fitness in the face of stressful environmental conditions, possibly conferring greater ecological breadth across their distribution (fitness homeostasis); and the hybrid “*Jack-and-Master*” (*JM*) strategy, where organisms are able to maintain average fitness in sub-optimal conditions and to opportunistically increase average fitness in optimal environments. *JM* species represent the “ideal” organisms [34], as they are considered more likely to have geographic ranges that can span diverse environments, are better able to respond to rapid environmental change, and are more likely to be highly invasive [20], [24]. Theory suggests that these plastic strategies can be expected to influence the shape, size and dynamics of species distribution [15], [21], [35], [36]. For example, a *JT* strategy occurring at range margins may be expected to sustain the size and the expansion of the species geographic range, whereas a *MS* strategy occurring at the range centre may help amplify the difference in population abundance between the centre and the margin of the range [6], [37]. The challenge is to determine whether this actually happens in the field.

**Figure 1:**
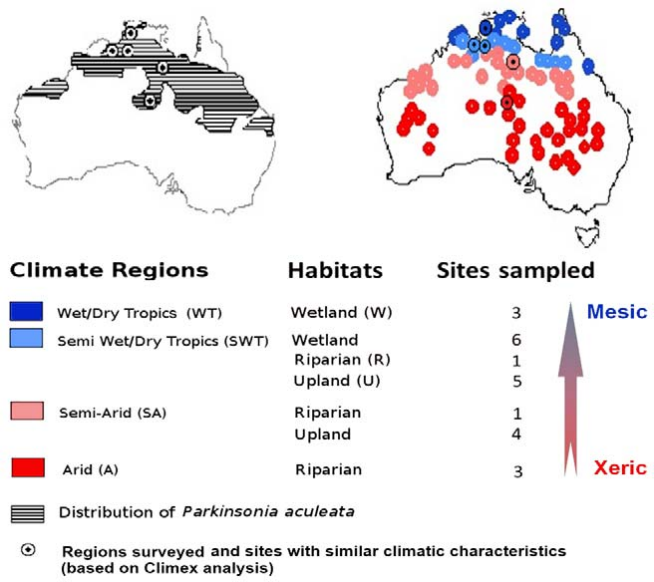
Spatial distribution of *P. aculeata* and survey design according to climate and habitat. **A**) Current distribution of *P. aculeata* across Australia (>800,000 km^2^) and the survey design across a 1,000 km rainfall gradient. **B**) Australian locations climatically similar to surveyed sites, as determined using climate-matching function in CLIMEX™ [70]. Within each climate region, populations were surveyed where possible between 2001 and 2007 in three habitat types (defined according to inundation patterns), for a total of 23 populations and more than 3,000 individuals at different life-stages.

**Figure 2:**
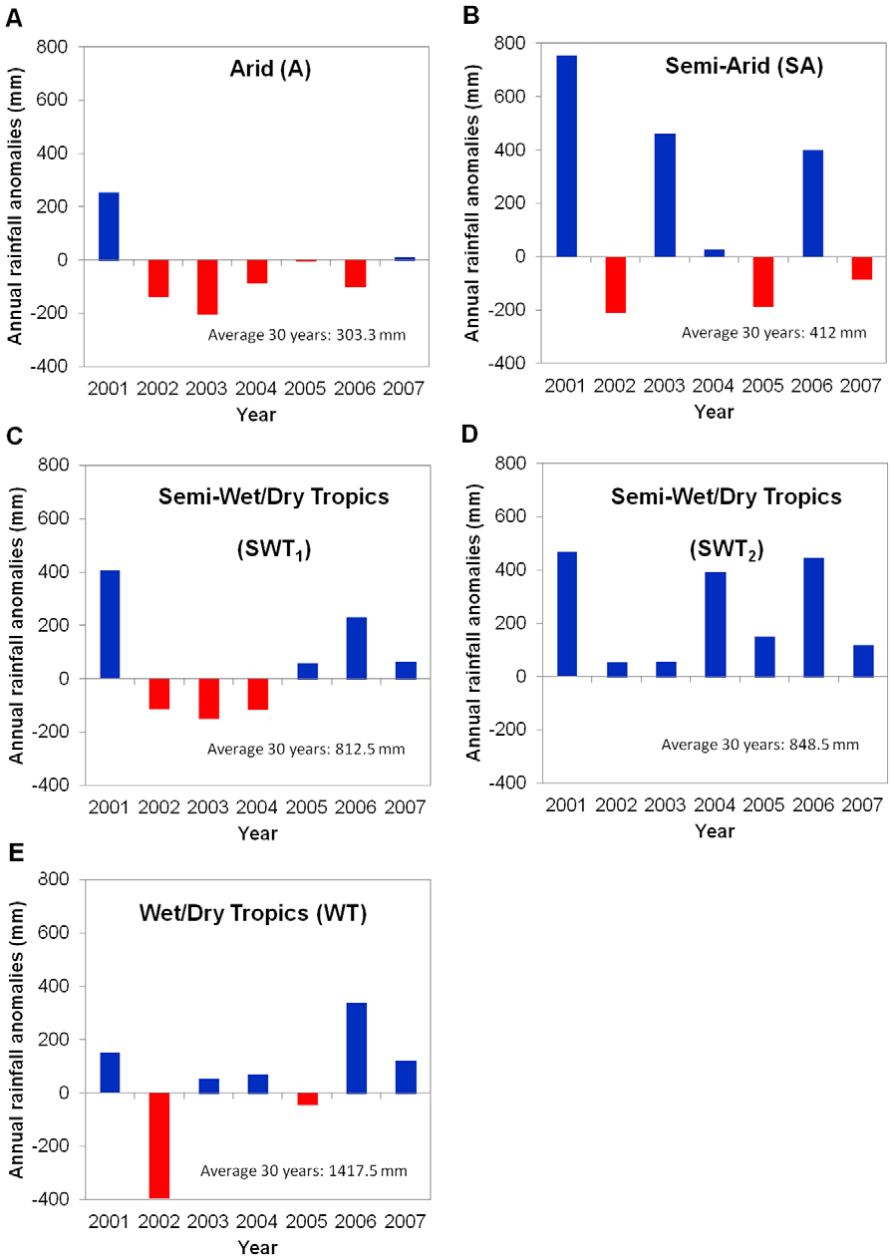
Rainfall anomaly histories for the different regions surveyed during the study period (2001–2007). The rainfall anomaly is the departure from average rainfall conditions (1961–1990). Details on the 23 sites and associated rainfall history can be found in Table S1.

**Figure 3:**
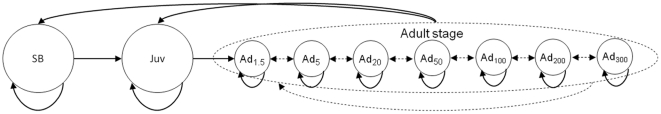
Life-cycle and life-stages of *Parkinsonia aculeata*. Nodes represent the three main demographic stages: seed bank (*SB*), juvenile (*Juv*: non-reproductive) and adult (*Ad*). Nodes (continuous lines) represent stages that can be stratified at an annual time step. The adult stage (dashed line) was stratified into 7 sub-stages according to canopy volume: *Ad_1.5_*, *Ad_5_*, *Ad_20_*, *Ad_50_*, *Ad_100_*, *Ad_200_*, *Ad_300_* m^3^: for instance in the volume class *A_1.5_*, trees have a volume between ]1.5 m^3^–5 m^3^], and *Ad_300_* have volume larger than 300 m^2^. Arcs that link nodes represent the flow of individuals that transit from one stage to another. Self-loops represent the capacity for individuals to delay the transition to the next stage.

In this study, we tested whether the geographic distribution structure and invasion success of the plant *Parkinsonia aculeata* L. (Fig. 1A) could be explained by a *JT*, a *MS* or a *JM* plastic strategy. Genetic variation in *P. aculeata* is low in Australia following its introduction, possibly as a single introduction from Venezuela, in the late 1800s [38]. However, it is now invasive in diverse habitats and its distribution spans a wide climatic gradient from the arid centre of Australia to the wet-dry tropics in the north. We tested whether phenotypic plasticity, acting through one or more of the proposed plastic strategies, contributed to this successful invasion. First we tested whether the allocation of resources between seed mass and seed number responded plastically to annual rainfall across a *ca.* 1000 km rainfall gradient (Fig. 1, Table S1) and between years for successions of abnormally wet and dry conditions (Fig. 2, Table S1). We then tested whether increasing seed mass resulted in higher seed and seedling survival rates. A matrix population model (see life-cycle in Fig. 3) [33], which incorporated the observed plastic response, was then used to test whether the average plastic response could influence the geographic distribution (shape, breadth, dynamic) through *JT*, *MS* or *JM* strategies, as predicted by theory. Finally, we assessed whether selection on the phenotypic response might result in further modifications in the species geographic distribution.

## Results

### Plastic mechanism

**Figure 4:**
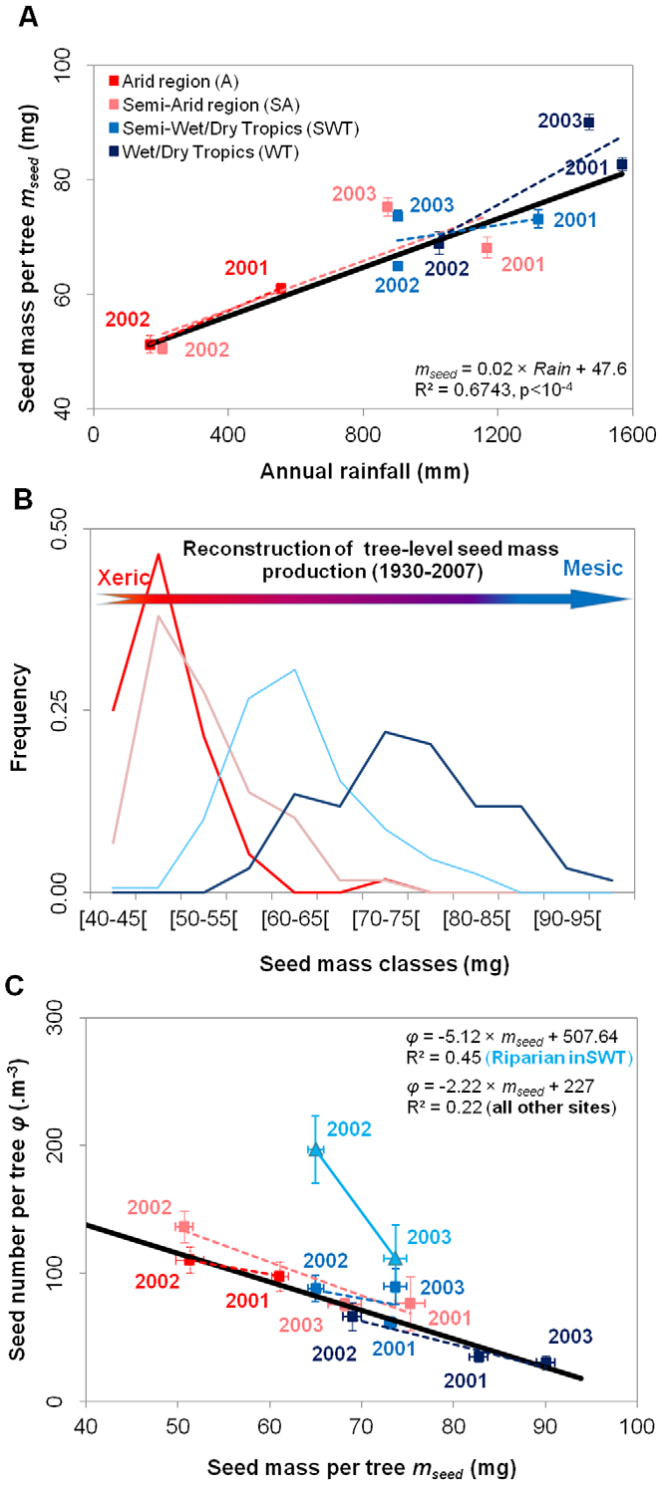
Tree-level plastic response between the mass of individual seeds and the number of seeds produced across the rainfall gradient. **A**) Reversible plastic tree-level response of the seed mass-seed number trade-off to rainfall. **B**) Reconstruction of seed mass distribution across the rainfall gradient from 1930 to 2007, using the observed plastic response. **C**) The most parsimonious tree-level trade-off between seed production and seed mass across the rainfall gradient. Nomenclature - colours correspond to climate regions, as in Figure 1A. Error bars represent s.e.

Seed mass at the individual tree level varied through space and time, and this was best explained as a plastic, reversible tree-level response of seed mass to rainfall. A hierarchical linear mixed-effect analysis which included trees and sites as random grouping factors (years *nested within* trees *nested within* sites) showed that variability in seed mass at the individual tree-level was greater between years at a site (s.d. = 7.8 mg) than between trees within a site (s.d. = 2.6E^−04^ mg), and was similar to variability between sites (s.d. = 6.4 mg). This random model was then compared with other models that incorporated combinations of climate, habitat, annual rainfall and time as fixed explanatory variables. The most parsimonious model showed that the variation in seed mass produced by each tree between years and between sites was best explained by changes in climate region, years and rainfalls (AIC = 1316, *wAIC* = 1). Habitat was the least significant factor (AIC = 1469, *wAIC* = 0). The average seed mass produced by each tree (*m_seed_*) responded linearly to rainfall (*m_seed_ = a*×*Rain*+*b*, *with a* = 0.02±9.10^−3^ (*t* = 10^−3^, *p*<10^−4^) *and b* = 47.6±0.97 (*t* = 50, *p*<10^−4^), *R^2^* = 0.6743), irrespective of whether rainfall varied through time within climate regions, or between climate regions across the environmental gradient (Fig. 4A). As a consequence, we observed for example that in the wet-dry tropics seed mass plastically varied annually, with seeds produced in the driest year (2002, Fig. 2E) being smaller than those produced in wetter years (2001, Fig. 2B) in the semi-arid areas (Fig. 4A). Similarly, seeds produced in the driest years in the semi-arid region (2002, Fig. 2B) were smaller than those produced in the wettest year (2001, Fig. 2A) in the arid region (Fig. 4A). A reconstruction of historical fluctuations in seed mass, based on local rainfall data since invasion commenced *ca* 1930 (using the relation between seed mass and annual rainfall), showed that the amplitude of the plastic response within the different climate regions may have been higher than observed from three years of observations, with individuals in the wet-dry tropics and arid regions frequently producing seeds with overlapping mass within their life time (c. 30 years) (Fig. 4B).

The average seed mass produced by each tree (*m_seed_*) was found to be linearly negatively correlated with the density of seeds produced (*ϕ*) by these same trees across the species distribution (Fig. 4C), such that *ϕ*  = −*α*×*m_seed_*+*β* (where the fecundity rate *f* = *ϕ* ×υ, and where υ is the tree canopy volume). The strength of the trade-off depended on habitat type and geographic location, with both the slope and intercept being higher in riparian (R) semi-wet tropics (SWT) (R^2^ = 0.44, n = 36, p<0.01, *α* = −4.17±1.28 and *β* = 404±82) than at other locations (R^2^ = 0.28, n = 196, p<0.001; *α* = −2.7±0.32 and *β* = 257±23). Riparian trees in the range centre (SWT) produced more seeds than expected given the rainfall in 2002, but seed mass was as predicted (Fig. 4A). Furthermore, the amplitude of seed mass/number plasticity in response to inter-annual change in rainfall was similar across climate regions, except in the arid margin where it was smaller (Fig. 4C).

### Adaptive significance of the plastic mechanism

**Figure 5:**
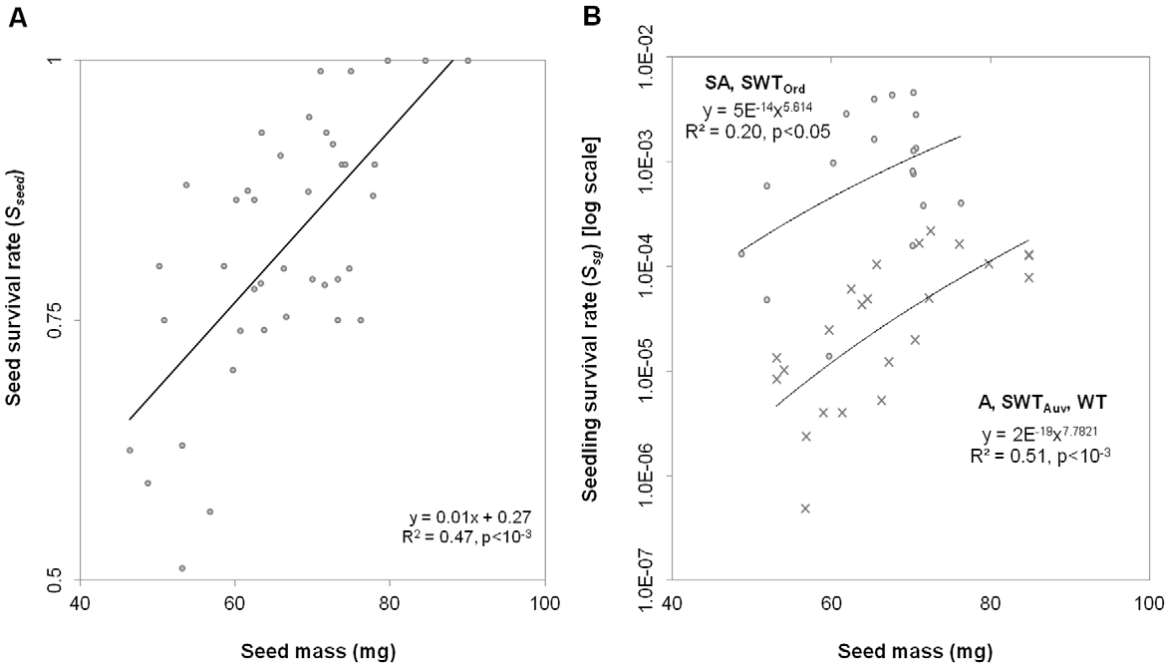
Benefits of plasticity on fitness components. A) on seed survival rate (*S_seed_*), and B) on seedling survival rate (*S_sg_*). A, SA, SWT, WT refer to the climate regions (see nomenclature in Fig. 1). For SWT, the Ord site (SWT_Ord_) and Auvergne site (SWT_Auv_) were analysed separately to account for the difference in *S_sg_*.

Seed mass was positively correlated with seed survival rate *S_seed_* (Fig. 5A) and seedling survival rate *S_sg_* (Fig. 5B). Seedling survival was also dependent on environmental conditions, with it being higher in some regions than others (Fig. 5B).

**Figure 6:**
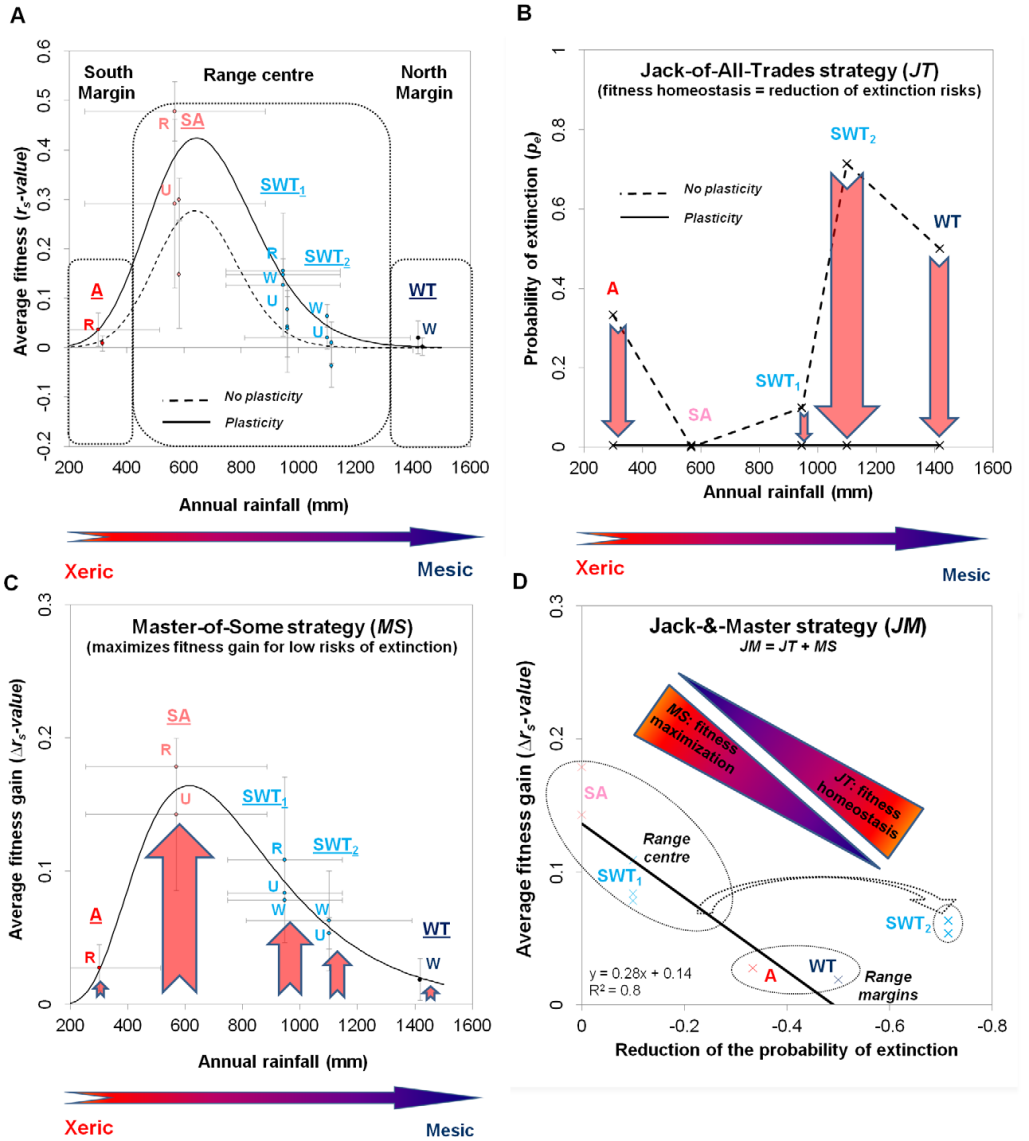
Average individual-level fitness benefits of plasticity. **A**) The log-normal distribution between annual rainfall and observed fitness (continuous line, average ± s.d) and fitness if there had been no plasticity (dashed line). **B**) The Jack-of-All-Trades (*JT*) strategy as predicted from the distribution between annual rainfall and observed probability of extinction (continuous line) and probability of extinction if there had been no plasticity (dashed line). **C**) Fitness gain provided by plasticity (average ± s.d). **D**) The Jack-&-Master (*JM*) strategy as predicted from the correlation between the reduction of probability of extinction and the average fitness benefits. The probability of extinction was calculated as the proportion of the populations with *r_s_*-value<0.

The trade-off between seed mass and seed number (Fig. 4C) and its relationship with seed and seedling survival rates (Fig. 5) were combined with other fitness components at a site scale per year (germination rate (Fig. S1), tree growth rate (Fig. S2) and survival rate (Fig. S3)) to estimate average fitness across the environmental gradient (Fig. 6). Average fitness (hereafter referred to as the *r_s_-value*) remained positive along the rainfall gradient, but was greatest in the range centre (Fig. 6A). The *r_s_-value* followed a lognormal distribution in response to the annual rainfall gradient (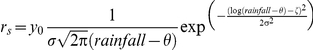; *R^2^* = 0.905, *χ^2^*/*df* = 0.0032; with *y_0_* = 195.88±1.44, *θ* = −577.5±136, *ζ* = 7.131±0.109, *σ* = 0.149±0.018).

Furthermore, simulations showed that trees in some populations would be maladapted *r_s_*<0 if they did not reversible compensate for a reduction in the number (or mass) of seeds produced by increasing seed mass (or seed number) (Fig. 6A: average effect of reducing seed number or seed mass *R^2^* = 0.84, *χ^2^*/*df* = 0.002; y_0_ = 100 (fixed), θ = −2024±597. ζ = 7.89±0.22, σ = 0.054±0.01). Reversible phenotypic plasticity contributed to fitness homeostasis, i.e. a *JT* strategy, by reducing the probability of extinctions for populations in the wet and dry margins and in the semi-wet tropics (SWT_2_) at the range centre (Fig. 6B). Reversible phenotypic plasticity also allowed for fitness maximization, i.e. a *MS* strategy, such that plasticity resulted in a higher fitness gain at the range centre than at the margins (Fig. 6C). A log-normal model (R^2^ = 0.94, χ2/df = 2.5E-4; with y0 = 109.41±0.17, θ = 66.41±136, ζ = 6.5±0.005, σ = 0.447±0.004) predicted that maximal average fitness benefits would occur in the semi-arid region (SA, 545 mm annual rainfall).

Both *JT* and *MS* strategies can co-occur across the species distribution, as predicted from the correlation between the reduction of extinction probability and the average fitness gain provided by plasticity (Fig. 6D). Trees tend to be more *MS* where the average fitness gain is high compared to the extinction probability gain (i.e., at the range centre), while trees will be more *JT* where the extinction probability gain is high compared to the average fitness gains (i.e., at the range margins). The semi-wet/dry tropic (SWT_2_) was an exception. Extinction risk was relatively high at that site despite it being in the range centre, and the *JT* strategy was therefore most pronounced. Altogether, the plastic reallocation between seed mass and seed number is a combination of *JT* and *MS* strategies across the species distribution, that is, a *JM* strategy.

### Selection pressure on the plastic mechanism

**Figure 7:**
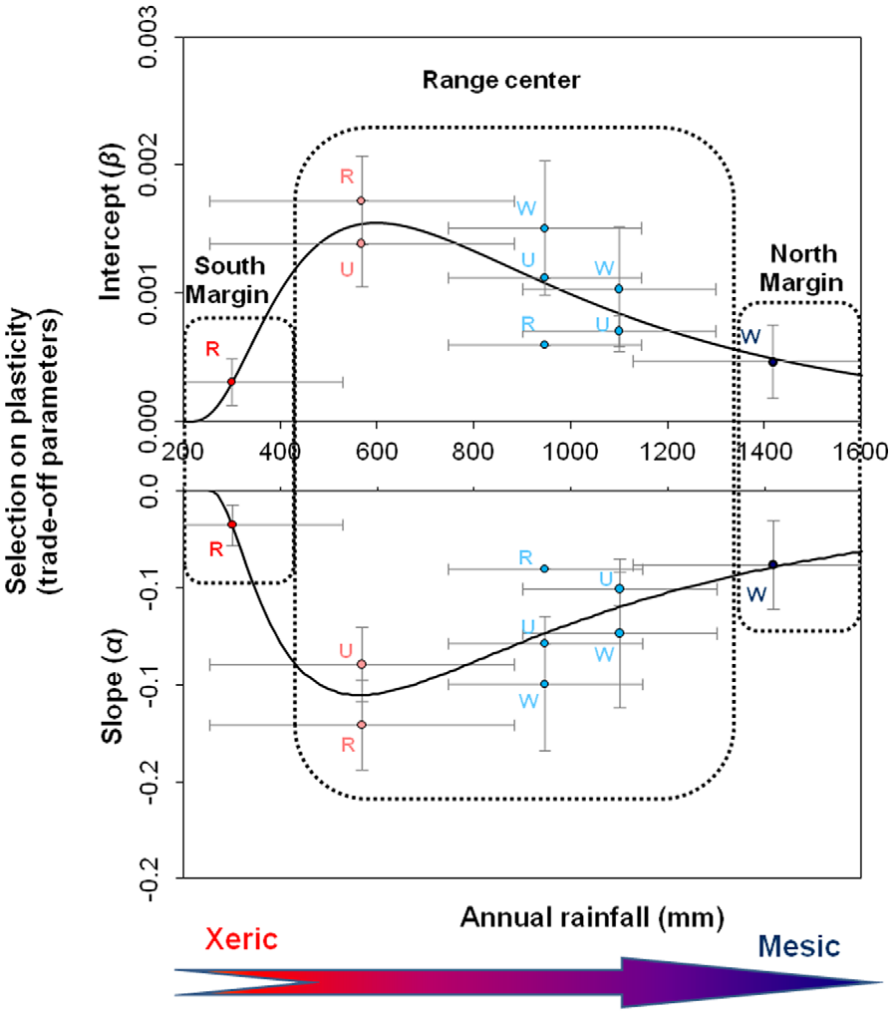
Selection pressure on the plastic trade-off across the annual rainfall gradient, calculated as the sensitivity of *average fitness* to potential change in the slope (*α*) and intercept (*β*) of the trade-off between seed mass and seed number. Error bars represent s.d. For the riparian habitat in the semi-arid hot and semi-wet tropical region, sensitivities are not stochastic and s.d. represents the variation between years at those sites.

Prospective sensitivity analysis suggests that plasticity itself is not fixed on an evolutionary time scale. This analysis suggests that both the slope *α* and intercept *β* of the trade-off between seed mass and number are under natural selection pressure (Fig. 7). Selection pressure on the slope was linearly correlated with the selection pressure on the intercept (Selection[α] = −59.9(±3.7)×Selection[β], R^2^ = 0.98, p<10^−6^), and both followed a lognormal distribution along the rainfall gradient (for selection on *α*: *R^2^* = 0.71, *χ^2^*/*df* = 5.10^−4^, with y_0_ = 120.7±2.08, θ = 244.8±3.9. ζ = 6.63±0.01, σ = −0.93±0.02; and for selection on *β*: *R^2^* = 0.73, *χ^2^*/*df* = 10^−7^, with y_0_ = 1.5±2.10^−4^, θ = 190.4±0.08. ζ = 6.54±5.97, σ = 0.73±2.10^−4^). Selection pressure is greatest at the range centre (for rainfall≈500–1100 mm), such that trees are expected to produce more seeds under ideal conditions while maintaining the same seed mass. This would result in a more pronounced MS at the range centre and maintenance of the *JT* strategy at range margins.

## Discussion

In this study we used a long-term, continental-scale field study of a highly invasive plant to provide the first empirical evidence for the adaptive significance of phenotypic plasticity across a species geographic distribution, and to provide insights into the plastic mechanisms underlying the “*Jack-and-Master*” strategy [20], [24]. Specifically, individual trees used a linear plastic trade-off between seed mass and seed number to maintain fitness across an environmental gradient (through a JT strategy) and maximize it where conditions were ideal (a *MS* strategy). A trade off between seed mass and seed number is a fundamental feature of plants which allows them to adaptively adjust the phenotype of their offspring in response to various environment characteristics [39], [40], [41]. Here we extend those findings to show that the “*Jack-and-Master*” strategy contributes to determining the limits, the shape and the dynamic of a species distribution. Furthermore, we show that the trade-off itself is under natural selection, which is expected to result in an even more pronounced “*Jack-and-Master*” strategy.

### Evidence for reversible plasticity across the species distribution

Demonstrating a phenotypic plastic response across an environmental gradient is contingent on excluding the possibility of local genetic adaptation [20], [24]. Cross-planting experiments are typically recommended to demonstrate plasticity on irreversible trait responses such as in stem size, plant/root biomass or seed production, especially for annual plants [20], [24]. However, they are generally impractical when looking at seed production of long-lived trees as it can take several years for reproduction to commence. We therefore used an alternative method that relies on tracking through time individual plants located across a spatially and temporally varying environment to test whether variability in seed mass was a reversible plastic response. This approach has previously been used to demonstrate reversible plastic responses in phenological development in response to climate variations or species interactions [42], [43], [44]. Our analysis showed that tree-level seed mass responded linearly to rainfall, irrespective of whether rainfall varied through time at a site, or between sites across an environmental gradient. We can therefore conclude that the observed seed mass response to rainfall is the result of the same reversible phenotypic plastic response across the species distribution, and not local adaptation.

### Plasticity and the overall adjustment of the life-cycle to environmental changes

Plants display a wide range of plastic responses in stressful environments, but there is uncertainty regarding the fitness benefits of coordinated plastic responses across their life-cycle [14], [30], [45], [46]. Matrix population models have been used to address this question on single plastic traits for various species with life cycles that have complex age or size structures [26], [29]. However, there is increasing recognition of the need to integrate more plastic relationships within life-cycle analyses [30], [47].

In our study, we show that *P. aculeata* uses two coordinated reversible plastic responses (i.e., the seed mass/number trade-off Fig. 4C) in order to adaptively respond to changes in rainfall, irrespective of whether rainfall varied through time at a site, or between sites across a 1000 km climatic gradient. A plastic response in the number of seeds produced (fertility rate) directly affects average fitness whereas plasticity in seed mass only affects average fitness through a positive relationship with seed and seedling survival rates (Fig. 5) (Caswell, 2001). Other less direct plastic relationships may play an adaptive role, although their contribution was not tested in this study. For instance, seed mass was positively correlated with germination rate *Germ* (i.e., the rate of dormancy release), the later also being passively induced by rainfall and heat (Fig. S2; see [48] for heat). Furthermore, seed production is delayed to larger tree sizes in the wet-dry tropics when compared to the arid region (estimated by multiplying seed production density [m^−3^] in Figure 4C by the volume *V* of the canopy [m^3^]).

These plastic responses are all individually well documented and are expected to help plants and animals to adaptively adjust to changing environments [39], [40], [41], [48], [49], [50], [51], [52], [53]. However, our study suggests that these individual plastic responses combine into a single plastic syndrome at the level of the life-cycle. Under very wet conditions (∼1400–1500 mm of annual rainfall), *P. aculeata* trees produce few-big-seeds that will germinate quickly and have higher survival rates though to the adult stage, but will delay seed production; while in very arid conditions (∼200–300 mm) trees produce many-small-seeds that will delay germination, and seed production will commence when plants are smaller. Under moderately wet conditions, an intermediate response can be observed, such that trees in riparian habitat within SWT produced many big seeds (Fig. 4C) and plants will start producing seeds when they are still relatively small (estimated by multiplying seed production density [m^−3^] in Figure 4C by the volume *V* of the canopy [m^3^]), probably in response to exceptionally good growing conditions [27].

In this study the integration of coordinated plastic responses across the life-cycle has provided important insights into the way organisms can adapt to new and changing environments. To extend our results, we propose that future studies should aim to capture the entire plastic syndrome of an organism across its life-cycle, synthesize it in a single integrated measure of plasticity (e.g. using measures already developed [21], [46], [54]), and estimate its overall adaptive significance in term of *average fitness* (using matrix population models or integral population models such as developed in [30]). By doing so on different species, we predict that general rules may be found on plasticity, which may help clarify how species optimise their life-cycle in response to environmental stresses [53].

### Plasticity and the structure of species geographic distributions

Phenotypic plasticity is suspected to play a role in determining the structure of species distributions. For instance, species distributions would be expected to be wider if plasticity helps maintain fitness under stressful environmental conditions [20], [21]. Furthermore, the shape of the species distribution may be affected if the fitness benefits of plasticity is higher in some locations than others [6], [37]. These expectations are supported by experimental studies that suggest that plastic species in some taxa have higher range breadth [15], [35] and that higher abundance of a species at the range centre can be related with higher plasticity on more traits [36].

Our study provides more direct support for these expectations. First, plastic responses resulted in a higher probability that *average fitness* remained positive across a broader set of climatic and habitat conditions (i.e., a *JT* strategy [20]), thereby broadening the species climate envelope into wetter climates in the north and drier climates in the south (Fig. 4A). Second, average fitness without plasticity was higher at the range centre than at the margins, but plasticity resulted in a further log-normal amplification of average fitness between the range centre and margins (Fig. 4B) (i.e., a *MS* strategy [20]). The measure of average fitness also measures the instantaneous and asymptotic growth rates of population abundance (see methods section), so plasticity also amplified the existing abundance centre of *P. aculeata* distribution [6], [37]. Our results therefore support previous expectations that plasticity can provide both greater robustness for species to respond geographically to climate change [17] than through genetic differentiation [4] (i.e, *JT* startegy) and greater responsiveness in their ability to expand their range more quickly when conditions are already good [11], [36], [37] (i.e, *MS* stategy). In our case, the invasive plant *P. aculeata* is able to use these two strategies and therefore behaves like a *JM* species.

### Plasticity and the dynamic of plant invasions

Plant invasions represent a special case of geographic range expansion, as it occurs in a new environment and is frequently a rapid process [7]. Phenotypic plasticity is expected to accelerate invasions at a large scale through different types of spatial strategies (*JT*, *MS* and *JM*: [20]). This is supported by experimental observations that introduced species show more plasticity than native species when individuals encounter new and more diverse conditions [24], [28], and by different spatial patterns of invasion according to the type of plastic strategy involved [20]. However, despite strong theoretical support, the role of plasticity on invasiveness in general [11], [12], and the different invasive strategies in particular [20], had not yet been demonstrated through overall population dynamics [22]. Here we mechanistically confirm at a large scale the critical role that temporal plasticity plays in quickly maintaining (through the *JT* strategy) or maximizing (through the *MS* strategy) the average fitness, respectively in optimal and sub-optimal conditions. Without the reversible plastic reallocation, the rate of invasion of *P. aculeata* would not have been as high in its range centre (*r_s_-values* maximized), and the current wet and dry range margins may have never been invaded (*r_s_-values*<0: Fig. 6A, 6B). Our results also confirm that large-scale invasions can occur across continental-scale environmental gradients without the need for genetic differentiation, and therefore without the lag-phases typically associated with even rapid genetic adaptations (Xu et al. 2010).

### “*Jack-and-Master*” and the evolution of species distributions

Reversible phenotypic plasticity, as a trait, can potentially evolve directly or indirectly in response to selection pressure [55], [56]. If plasticity evolves differently in different locations of the species distribution during or after the invasion phase [17], [20], [45], the resulting spatial heterogeneity in average fitness benefits are expected to impact the shape of the geographic distribution [37]. However, no study provides mechanistic predictions for this phenomenon. Here we show that the selection pressure on the parameters describing the trade-off between seed mass and number (slope and intercept) is expected to increase *average fitness* more at the range centre than at range margins. Therefore, selection on plasticity itself may result in a stronger *MS* strategy at the range centre, whereas the *JT* strategy may already be near its evolutionary limit at the margins. Evolution of the *JM* strategy may therefore contribute more in reinforcing the invasion process within the current climate envelope by amplifying the abundant centre shape of distribution, rather than expanding its existing range limits. This is not to say, however, that further range expansions are not possible as the result of selection on other fitness components.

### Implications for modelling species invasions and distributions

Current explanations and predictions of species invasiveness and geographic distributions rely largely on species distribution models (SDMs: [57]). However, such models do not consider possible effects of phenotypic plasticity at large scales [24], [47], [58], and especially the underlying plastic strategy (*JT*, *MS* or *JM*). Our results suggest that SDMs that explicitly consider reversible plastic responses may predict very different species range dynamics compared to models that only consider genetic differentiation. For example spread or shift rates will be faster than observed through genetic differentiation, and will be less sensitive to environmental perturbations at different scales. General rules on the role of plasticity in explaining the underlying process of invasion and species range dynamics may emerge by replicating this type of study on other species, other types of plastic traits and at different temporal and spatial scales.

## Materials and Methods

### Biology of *P. aculeata* in Australia

*P. aculeata* is invasive in Australia across an extraordinarily broad geographic range (Fig. 1), from upland to wetland habitats and from arid to tropical climates [59], [60]. Its life-cycle consists of several life-stages (Fig. 2): seed bank (*SB*), seedlings (*Sg*), juveniles (*Juv*) and adults (*Ad*). Tree growth, the number of seeds produced, and seed germination (*Germ*) predominantly occurs in the warm to hot months of the year. Indehiscent pods (containing 1 to 9 seeds) fall directly under the parent tree and release most seeds by the end of summer. *SB* was defined to include all live seeds present in both the soil and the litter layers. Seeds have hard-seeded dormancy from which they are released by wet, warm to hot conditions. Most are released from dormancy and either germinate or die within the first 1–2 years. Germination resulted in seedlings (<20 cm tall) that become small juveniles (20–100 cm tall) by the first winter census. Seedling survival rates (*S_sg_*), i.e. the proportion of new seedlings that survived and established as juveniles by their first winter census, was included within the transition rate between *SB* and *Juv*. Small juveniles became large juveniles (100–150 cm tall) before becoming adults (>150 cm tall). After establishment, juveniles take at least one year to mature. Adults grow continuously to over 7 m in height and 10 m in width, with total seed production increasing proportionally with canopy volume, but they rarely live longer than 30 years under natural conditions. Adults were stratified into seven sub-stages *i* (defined by canopy volume *V*: see Fig. 3) to provide better resolution of size-dependent fitness components such as annual growth rate (*G_Vi_*), survival rate (*S_Vi_*) and total number of seeds produced (the fecundity rate *f_Vi_*).

### Survey design

No specific permits were required for the described field experiments. We selected 23 sites in Australia's Northern Territory along a 1000 km climatic gradient spanning four climate regions (arid, semi-arid, semi-wet/dry tropics, wet/dry tropics). Sites were chosen to best represent the range of habitat types within each climate zone (upland, wetland and riparian) (Fig. 1A, 1B), and that could be accessed year-round. The surveyed populations had few or no other competing plant species, and had sufficient canopy openings for continued invasion and to minimize density-dependent effects. We selected sites to include at least 10 healthy large adults (>200 m^3^). Where there was a discrete population, the entire population was included within the site. Surveys at all life-stages were conducted from 2001 to 2007 annually during winter (June-Sept) when conditions were cool and dry and populations were relatively static. Some sites were terminated prematurely (and in some cases replaced with new sites) following major disturbance events. The site characteristics can be found in Table S1. All seedlings (<20 cm) and small juveniles (20–100 cm) were systematically censused from 2001 to 2007. All trees above 1 m in height were individually tagged during the first census, or as they reached that size in subsequent censuses. For each tagged plant we recorded its fate (alive or dead), height, and canopy width (along both axes). Canopy volume was used to track growth by assuming canopy volume as a spheroid. Seed bank density (*SB*) was estimated during each census using four randomly placed soil cores (area = 38.5 cm^2^, 5 cm deep) placed under each of five randomly selected large adult trees.

### Measurements of the mass and number of seeds produced per tree and estimation of the plastic response

The number of seeds and the average seed mass per tree (calculated from 100 randomly selected seeds) were measured annually between 2001 to 2003, by placing litter traps under the canopy of ten randomly selected large adult trees (≥100 m^3^ in canopy) within each of the 22 sites. Annual number of seeds produced per tree (fertility rate ***f_V_***) was estimated by multiplying seed rain density between the two censuses with total tree canopy area. ***f_Vi_*** represents the total number of seeds produced by a tree of a volume-class *v*. Because fecundity was estimated on the big trees, estimates were extrapolated when necessary for smaller adult volume classes (volume classes [1.5–5 m^3^[, [5–20 m^3^[and [20–50 m^3^[), using exponential regression of fecundity against tree volume for every site and year. Seed production at some sites was greatly reduced in trees that severely affected by a soil-borne pathogen [59], and these trees were therefore excluded when estimating the plastic response and other life-history traits [14]. We used tree-level estimates of seed mass and seed number (seeds per canopy area) to model the average trade-off between seed mass and number across the species distribution. We then modelled its relation with rainfall in the year preceding seed production.

The reversibility of the trade-off between seed mass and seed number was tested by determining whether the average seed mass at the individual tree level varied more through time than between trees within and between sites [14], [61]. We used hierarchical mixed effect models [62] to compare the variance of seed mass i) per individual through time (2001–2003) at a site scale, ii) between individuals per site and iii) between the 22 sites across space. The 22 sites and the individual trees (*nested within* the sites) were both used as random grouping factors for the response variables. The different years were nested within the individuals and the temporal variance represented the residuals. We then compared this random model with other models that incorporated combinations of climate, habitat, annual rainfall and time as fixed explanatory variables. The best model was used to estimate and compare the spatial and temporal variance of seed mass and to estimate seed mass under different spatial and temporal conditions. The package *nlme* from *R-cran* was used for the hierarchical mixed affect analysis (*R* Development Core Team, 2006).

### Estimating the fitness benefits of seed mass increase on seed and seedling survival rates

Seed mass is expected to be positively correlated with seed survival and seedling survival rates [41]. Seed survival rate (***S*** *_seed_*) was defined as the proportion of seeds that are still alive in the following year (i.e. as dormant seeds or as new germinants). We estimated annual seed survival between 2001 to 2003 using a standardized laboratory test [48], [63] on seeds sampled each year from the seed rain study (see above).

Seed dormancy-release and germination is very sensitive to local environmental conditions so annual seed germination rates could not be estimated in the laboratory. Seed germination was therefore estimated indirectly from seed density in the current winter ***N*** *^SB^_[t]_*, the density of seeds present in the seed bank of the previous winter ***N*** *^SB^_[t−1]_* that were still alive in the current year (***S_seed_***_* [t−1,t]*_), and density of seeds that were produced in the interceding year that were still viable and remained dormant in winter (***f*** *_V [t−1/2]_*×(1−***Germ***[t−1, t])). Germination between two time steps was estimated as follows:
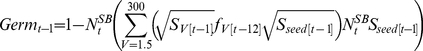
(1)with ***V*** indicating the volume classes of the tree, and with
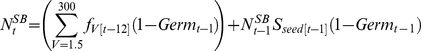
(2)


We estimated the new germinants from the number of trees that reproduced, the number of seeds produced per tree, seed survival and the seed germination rate. Then, annual seedling survival rate (*S_Sg_*) for the period 2001–2007 was estimated as the proportion of new germinants that reach the juvenile stage.

### Estimating average fitness

The average fitness at an individual tree level was estimated using a matrix population model after first estimating individual fitness components for plants of different sizes (growth, survival and fertility rates).

Juvenile and adult growth and survival rates at each site were calculated empirically from annual census data from individually tagged trees (large juveniles >1 m tall) and un-tagged small juveniles (<1 m). Only healthy plants were used to estimate growth rates in order to determine the undisturbed plastic response of trees [14]. As a consequence, plants that were dying or killed by a dieback phenomenon attributed to soil-borne pathogens [59], [60] were excluded (24.5% of all plants, ranging from 0% in the semi-arid region (SA) to 32.2% in the semi-wet/dry tropics SWT_2_), as were plants killed by severe frost in arid Australia (8.2%).

Juvenile and adult growth and survival rates, and seedling survival rates, were estimated for a maximum of six years between 2001 and 2007. Where possible, juvenile and adult growth and survival rates were estimated per site and year. Seed survival rate, germination rate, seed mass and seed number (i.e. fertility) were only estimated from 2001 to 2003. These values were therefore extrapolated through to 2007 using empirical relationships between seed mass and annual rainfall, seed mass and seed survival, germination and rainfall, and seed mass and seed number (see results).

The trade-off between seed mass and number, and its relation with seed and seedling survival rates, were integrated together with other fitness components (i.e., germination, tree growth, survival and fertility rates) within a (*9×9*) demographic transition matrix *A* [33]:
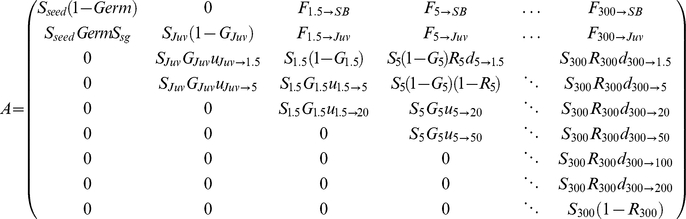
where matrix *A* projects the fate of the individuals between the 9 life-stages between two time steps *t−1* to *t*. In matrix *A*, two annual fertility rates are implemented in order to represent the different fates of a seed from *t−1* to *t*. 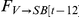 represents the actual rate that a tree at a particular volume class ***V*** at time *t−1* is alive half way between censuses *t−1/2* (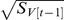) and produces a number of seeds (*f_V[t−1/2]_*) that will survive from *t−1/2* to *t* (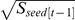) and stay dormant in the seed bank (1−*Germ*):
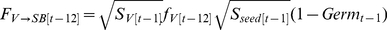
(3)

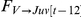
 represents the rate of the seeds produced that will germinate and survive as seedlings (*S_sg_*) from *t−1/2* to *t* and therefore establish as *Juv* at time *t*:
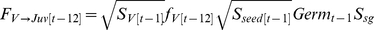
(4)


Following the Lande's theorem [31], [32] and p.284 and 437 in [33], we can use the log of the stochastic population growth rate (log ***λ_s_*** called *r_s_* value in the text) of the transition matrix ***A***, in order to measure the *average fitness* of an individual during its entire life-cycle under certain conditions [64], [65]. The necessary conditions for using the *r_s_-value* as an average measure of fitness are: no large genetic variation between individuals and populations, no density dependence, no resource limitation, and that the population is surveyed at the stable stage distribution. Genetic variation in *P. aculeata* is low in Australia [38]. Density-dependence and resource limitation were satisfied by site selection. The stable-stage distribution was tested and confirmed using a *χ^2^-test* (Table S2). This test compared the observed population structure with the expected stable-stage distribution determined from the right eigenvector of the demographic matrix ***A*** [33]. The package *Popbio* from *R-cran* was used for all matrix population analyses (***R*** Development Core Team, 2006). For every site we estimated a transition matrix ***A*** containing the fitness components averaged between 2001 and 2007 and estimated log ***λ_s_***, following eqn. 14.72 in [33]:
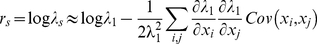
(5)where *λ_1_* represents the first eigenvalue of the matrix *A* (i.e., the deterministic population growth rate), 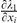 and 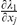 represent the sensitivity of the first eigenvalue to changes in the fitness components *x_i_*, and *x_j_*, and *Cov*(*x_i_, x_j_*) represents the variance-covariance matrix containing the variance and covariance between every pair of fitness components (*x_i_, x_j_*) from 2001 to 2007.

### Estimating average fitness benefits of plasticity across the species distribution

The effect of the observed level of phenotypic plasticity on average fitness across the rainfall gradient was tested. We first calculated average fitness (*rs-value*) for each site using the observed trade-off between seed mass and seed number for that site. We then compared this result with average fitness assuming that there was no reallocation between seed mass and seed number across the moisture gradient (i.e., no plastic reallocation). This was done using the lowest average seed mass (i.e., 48.7 mg), or lowest fertility from where seeds were biggest (in the wet-dry tropics). We compared the *r_s_-values* across the rainfall gradient (with or without plasticity) to assess whether plasticity allowed some populations to maintain positive *r_s_-values* (JT), to increase already positive *r_s_-values* (MS), or both (JM). We then tested whether the geographic pattern of JT,MS or JM strategies (i.e. the gain in *r_s_-values* provided by plasticity) could be predicted by the rainfall gradient. For this analysis, we fitted a non-linear model of distribution using the software SciDavis and the Scaled Levenberg-Marquardt algorithm with a tolerance 10^−4^ (SciDavis development team, 2010).

### Estimating selection pressure on plasticity across the species distribution

Multivariate linear regression analysis can be used to estimate the selection pressure on plastic morphological traits when few fitness components are measured [66], [67], [68], [69]. However, when all the fitness components through the life-cycle of a species are estimated, we can use the properties of the transition matrix ***A*** to mechanistically assess whether the strength of the plastic reallocation itself (i.e., slope *α* and intercept *β* of the trade-off) is under selection pressure at an individual tree level, with regard to the *r_s_-values*. For this type of analysis, we used a lower-level prospective sensitivity analysis of the *r_s_-values* (equ. 14.105 p.407 in [33]). As we used the average estimate of the trade-off between seed mass and number, we approximated the sensitivity of the *rs-value* (log*λ_s_*) to changes in the slope *α* and intercept *β* by equ. 14.107 p.407 in [33], such that:
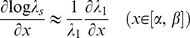
(6)We then analyzed whether the level of selection pressure could also be predicted by the rainfall gradient, by fitting a non-linear model with the software SciDavis.

## Supporting Information

Table S1 **Details of the sites surveyed.** * The closest climate station to the field site **(** http://www.bom.gov.au/climate/data/ **).**
(XLS)

Table S2 **Chi-square test for the stable population structure.** The test shows the difference between the observed population vector and the predicted stable population vector (i.e., the right eigenvector of the transition matrix). These vectors give the relative proportion of individuals at the different life-stages (SB, *Juv*, *Ad_1.5_*, *Ad_5_*, *…*, *A_d 300_*). Because the fraction of seeds in the seed bank (SB) was much higher than the fraction of individuals at all other life-stages combined (more than 0.95), we tested the difference with and without the seed bank.
(XLS)

Figure S1 **Correlation between site-level annual rainfall and germination rate (Germ).** The estimated germination rates confirmed previous experimental results.
(TIF)

Figure S2 **The empirical relationship between canopy volume (** ***V*** **) and plant growth rate (** ***G*** **) in different climate regions.** Where possible, points represent growth rate values per site and year for a given climate region.
(TIF)

Figure S3 **The empirical relationship between canopy volume (** ***V*** **) and plant survival rate (** ***S*** **) in different climate regions.** Where possible, points represent survival rate values per site and year for a given climate region.
(TIF)
