# Supplementary figures and images for: Phenotypic Plasticity Influences the Size, Shape and Dynamics of the Geographic Distribution of an Invasive Plant
Source: PLoS One. 2012 Feb 27;7(2):e32323. doi: 10.1371/journal.pone.0032323 (PMC3288080; doi:10.1371/journal.pone.0032323)

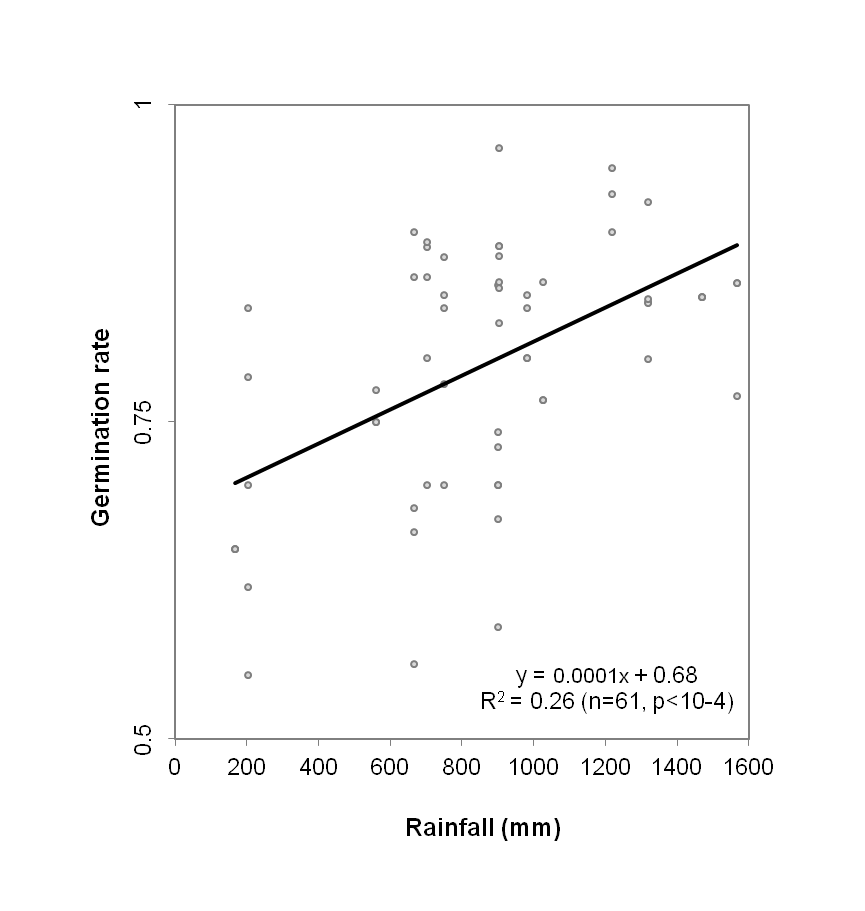

Supplement: Figure S1 — Correlation between site-level annual rainfall and germination rate (Germ). The estimated germination rates confirmed previous experimental results. (TIF) [file pone.0032323.s003.tif]

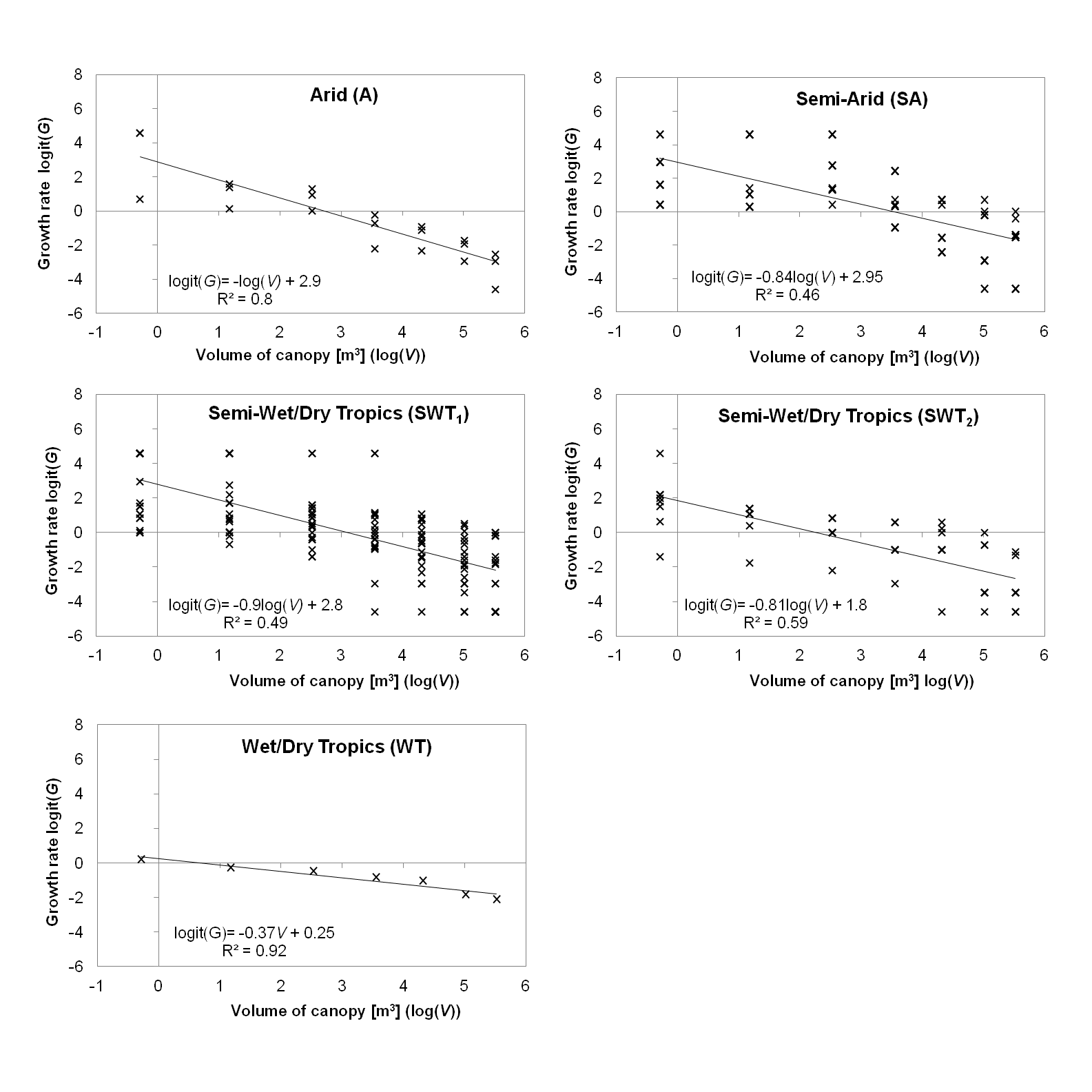

Supplement: Figure S2 — The empirical relationship between canopy volume ( V ) and plant growth rate ( G ) in different climate regions. Where possible, points represent growth rate values per site and year for a given climate region. (TIF) [file pone.0032323.s004.tif]

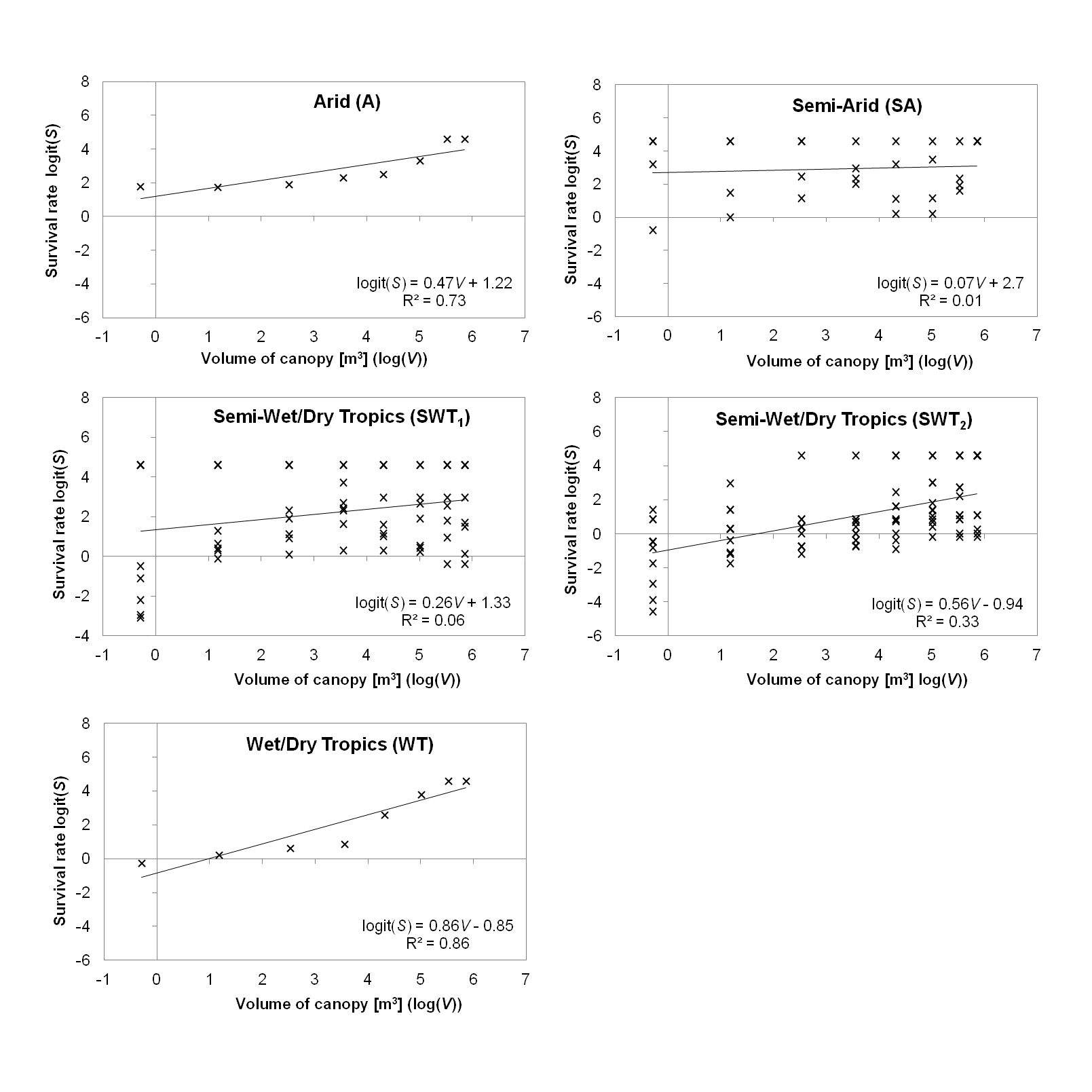

Supplement: Figure S3 — The empirical relationship between canopy volume ( V ) and plant survival rate ( S ) in different climate regions. Where possible, points represent survival rate values per site and year for a given climate region. (TIF) [file pone.0032323.s005.tif]
